# Multimodal exercise training to reduce frailty in people with multiple sclerosis: study protocol for a pilot randomized controlled trial

**DOI:** 10.1186/s40814-024-01496-2

**Published:** 2024-04-22

**Authors:** Tobia Zanotto, Danya Pradeep Kumar, Abbas Tabatabaei, Sharon G. Lynch, Jianghua He, Trent J. Herda, Hannes Devos, Ramkumar Thiyagarajan, Lee Chaves, Kenneth Seldeen, Bruce R. Troen, Jacob J. Sosnoff

**Affiliations:** 1 Department of Occupational Therapy Education, School of Health Professions, University of Kansas Medical Center, Kansas City, KS 66160 USA; 2 Mobility Core, University of Kansas Center for Community Access, Rehabilitation Research, Education and Service, Kansas City, KS USA; 3 Landon Center on Aging, University of Kansas Medical Center, Kansas City, KS USA; 4 Department of Physical Therapy, Rehabilitation Science, and Athletic Training, School of Health Professions, University of Kansas Medical Center, Kansas City, KS USA; 5 Department of Neurology, School of Medicine, University of Kansas Medical Center, Kansas City, KS USA; 6 Department of Biostatistics and Data Science, University of Kansas Medical Center, Kansas City, KS USA; 7 Department of Health, Sport, and Exercise Sciences, University of Kansas, Lawrence, KS USA; 8 Division of Geriatrics, Department of Internal Medicine, School of Medicine, University of Kansas Medical Center, Kansas City, KS USA; 9 Research Service, Kansas City Veterans Affairs Healthcare System, Kansas City, MO USA

**Keywords:** Frailty, Multiple sclerosis, Exercise, Resistance training, Rehabilitation

## Abstract

**Background:**

Frailty, a syndrome characterized by decreased reserve and resistance to stressors across multiple physiologic systems, is highly prevalent in people living with multiple sclerosis (pwMS), independent of age or disability level. Frailty in MS is strongly associated with adverse clinical outcomes, such as falls, and may aggravate MS-related symptoms. Consequently, there is a pressing necessity to explore and evaluate strategies to reduce frailty levels in pwMS. The purpose of this pilot randomized controlled trial (RCT) will be to examine the feasibility and preliminary efficacy of a multimodal exercise training program to reduce frailty in pwMS.

**Methods:**

A total of 24 participants will be randomly assigned to 6 weeks of multimodal exercise or to a waitlist control group with a 1:1 allocation. PwMS aged 40–65 years and living with frailty will be eligible. The multimodal exercise program will consist of cognitive-motor rehabilitation (i.e., virtual reality treadmill training) combined with progressive, evidence-based resistance training. At baseline and post-intervention, participants will complete the Evaluative Frailty Index for Physical Activity (EFIP), measures of fall risk, and quality of life. Frailty-related biomarkers will also be assessed. In addition, the feasibility of the multimodal exercise program will be systematically and multidimensionally evaluated.

**Discussion:**

To date, no RCT has yet been conducted to evaluate whether targeted exercise interventions can minimize frailty in MS. The current study will provide novel data on the feasibility and preliminary efficacy of multimodal exercise training as a strategy for counteracting frailty in pwMS.

**Trial registration:**

ClinicalTrials.gov, NCT06042244 (registered in September 2023).

**Supplementary Information:**

The online version contains supplementary material available at 10.1186/s40814-024-01496-2.

## Background

Frailty is a biological syndrome of decreased reserve and resistance to stressors arising from cumulative declines across multiple physiologic systems [1]. Frailty is very common in individuals living with multiple sclerosis (MS), regardless of age and level of disability. People with MS (pwMS) have up to a 15-fold higher risk of being frail compared to age-matched individuals living without MS [2–4]. Frailty within MS is strongly associated with common MS-related problems such as walking deficits [5] and with adverse clinical outcomes such as falls [6]. In addition, pwMS become frail at a younger age compared to individuals without MS [4]. These observations highlight the critical need to identify and evaluate strategies for counteracting frailty in pwMS. Reducing frailty levels could have a significant impact on improving the health of pwMS.

There is considerable evidence that targeted exercise interventions can improve physiological function in pwMS [7]. The general benefit of exercise in pwMS highlights the possibility that a well-designed exercise program may be a viable strategy to reduce frailty in this population. This possibility is further bolstered by the numerous randomized clinical trials (RCT) that reported promising findings in terms of frailty reduction following exercise interventions in older adults. Recently, we completed a 6-week RCT focusing on virtual-reality treadmill training (i.e., cognitive-motor training) in over 100 pwMS living with moderate frailty and found an improvement in several hallmarks of frailty, such as gait speed, cognition, and depression, following the intervention [8]. Importantly, the virtual-reality treadmill training used in our previous investigation was originally conceived to reduce the risk of falling, a significant corollary measure of frailty [9]. In addition, an advantage of virtual-reality treadmill training compared to treadmill training alone is that it targets both motor and cognitive dysfunction, which are both very common in pwMS [10] and implicated in the etiology of frailty [11, 12]. However, an important limitation of our previous study is that it was not specifically designed to target frailty. Particularly, although successful at enhancing several key aspects of frailty (e.g., gait and cognition), our intervention did not include resistance training (RT), a crucial component of exercise programs aiming to minimize frailty in geriatric populations [13–15].

Progressive resistance training (RT) is widely regarded as an active ingredient to counteract frailty and frailty-related problems in geriatric populations and in people living with chronic diseases by restoring muscle strength and promoting anabolic effects [16, 17]. Importantly, studies involving pwMS have shown that, in addition to improving muscle strength [18, 19], RT can also improve MS-related signs and symptoms such as fatigue, mood, sleep quality, cardiac autonomic function, and markers of inflammation [18, 20–23]. It has been proposed that RT combined with other exercise modalities such as gait and balance training (i.e., multimodal exercise) may represent the best strategy to improve the hallmarks of frailty in frail individuals [24]. To date, however, no study has yet been conducted to explore whether multimodal exercise training or exercise interventions in general can reduce frailty levels in pwMS.

The purpose of this study will be to address this critical research gap by exploring the feasibility and preliminary efficacy of a multimodal exercise training program (compared to a passive waitlist control group) to reduce frailty in pwMS. Feasibility will be comprehensively and multidimensionally assessed to inform the development of a larger RCT. In addition, we hypothesize that participants in the multimodal exercise group will have a greater reduction in frailty [25] than participants in the control group.

## Methods

### Study design

**Fig. 1:**
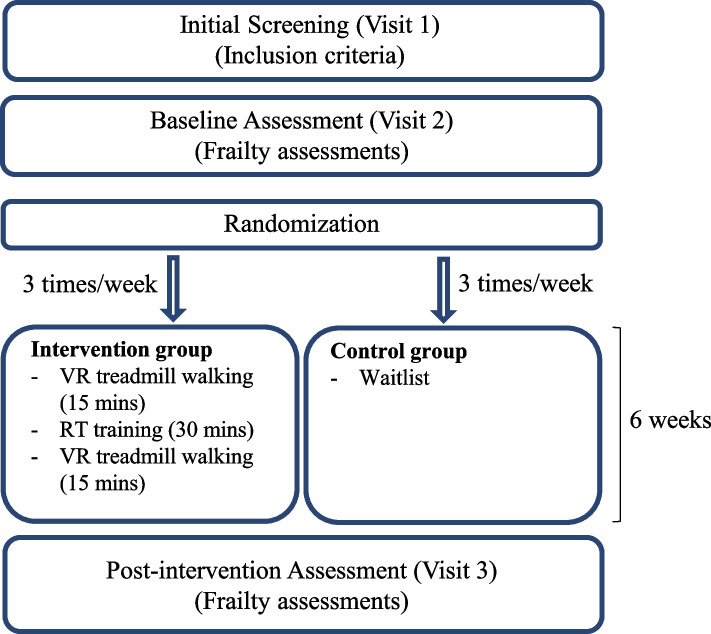
Study design. RT, resistance training; VR, virtual reality

The present study will be a pilot two-parallel group assessor-blinded randomized controlled trial (RCT) study (ClinicalTrials.gov registration number: NCT06042244). After the initial screening visit (Visit 1), a total of 24 ambulatory pwMS living with frailty will undergo the baseline assessment (Visit 2). Participants will be randomly allocated to one of two groups using a 1:1 randomization sequence: an interventional group consisting of 6 weeks of multimodal exercise training (*n* = 12) or a waitlist control group (*n* = 12). Participants will then complete the post-interventional assessment (Visit 3), between 4 and 7 days following the last training session (Fig. 1). The ethics of the study conform with the 1964 Declaration of Helsinki and its later amendments and have been reviewed and approved by the University of Kansas Medical Center Institutional Review Board (STUDY00149742).

### Screening, eligibility criteria, and recruitment

People with MS (pwMS) receiving medical care at the University of Kansas Medical Center will be comprehensively screened to ascertain their eligibility for participation in the study (Visit 1). The treating neurologist and a trained research assistant will identify potential individuals who meet the predefined inclusion and exclusion criteria outlined in Table 1. To screen the potential participants for frailty and cognitive impairment, we will use the Fried phenotype [26] and the Mini-Cog questionnaire [27], respectively. The Fried phenotype evaluates five frailty components, namely, unintentional weight loss, exhaustion, weakness, slowness, and inactivity, using established cutoffs [26]. Individuals meeting at least three of these five components will be classified as frail and will be eligible for study participation. Additionally, individuals achieving a score above three on the Mini-Cog evaluation will be considered eligible [27]. Participants will provide written informed consent to the research assistant before taking part in the study.

**Table 1:** Inclusion and exclusion criteria

**Screening**	**Inclusion criteria**	**Exclusion criteria**
In-clinic screening	• Age 40–65 years • Confirmed MS diagnosis by a neurologist • EDSS ≤ 6.0 • Fluency in spoken and written English	• The presence of other clinically important neurological conditions including Parkinson’s disease and epilepsy • Active psychiatric problems • The presence of unstable cardiovascular conditions, arthritis of the lower limb, acute lower back or lower limb pain, rheumatic and/or severe orthopedic problems that may interfere with resistance training
Onsite screening	• Frailty as defined by the Fried frailty phenotype	• Unable to walk 10 m unassisted • Severe cognitive impairment (Mini-Cog < 3)

*EDSS* Expanded Disability Status Scale

### Sample size

For this pilot study, we will enroll 24 ambulatory pwMS living with frailty and randomize them equally into the intervention and control groups. This sample size meets the sample size rules of thumb of 12 per group for two-armed pilot trials [28]. We expect ≥ 80% retention; that is, about 10 subjects/groups will complete the study. Particularly, a sample size of 24 will enable us to estimate a retention rate of 80% to be within a 95% Clopper-Pearson exact confidence interval of (0.59, 0.93). In addition, based on current prevalence estimates of frailty in pwMS [2, 6], we anticipate that at least one-third of subjects identified during the in-clinic screening phase (Table 1) will meet the frailty criteria evaluated during the onsite screening phase. Therefore, if we identify 72 potentially eligible subjects during the recruitment process (i.e., 24/0.33), we will be able to estimate a conservative recruitment rate of 25% to be within a 95% asymptotic confidence interval of +/−10%.

### Data collection methods and outcome measures

The preliminary efficacy and feasibility outcomes (primary, secondary, and tertiary) of the study are fully summarized in Table 2. Once participants meet the screening criteria (Visit 1), they will attend the baseline assessment (Visit 2). The baseline assessment will encompass a blood test (i.e., frailty-related biomarkers) and a comprehensive assessment of frailty (including corollary measures such as quality of life and fall-risk). These assessments will be performed in the Landon Center on Aging at the University of Kansas Medical Center. The same procedures will be repeated for the post-training assessment (Visit 3), which will take place between 4 and 7 days following the last training session.

**Table 2:** Primary, secondary, and tertiary study outcomes

**Information collected from all participants**	**T**_**O**_	**T**_**1**_	**In**	**T**_**2**_	**O**
**Sociodemographics**					
Age, gender, marital status, race, height, weight, education, and occupation	✓				D
**Frailty status**					
Fried Frailty Status Assessment	✓				D
**Cognitive status**					
Mini-Cog	✓				D
**Comprehensive Frailty Assessment**					
Evaluative Frailty Index for Physical Activity (EFIP)		✓		✓	P
**Fall-risk screening**					
Physiological Profile Assessment (PPA)		✓		✓	S
**Quality of life**					
Multiple Sclerosis Quality-of-Life questionnaire (MSQoL-54)		✓		✓	S
**Blood test**					
Blood biomarkers		✓			T
**Safety and feasibility outcomes**					
Pain (numeric pain rating scales)		✓	✓	✓	P
Adverse events — defined by the NIH		✓	✓	✓	P
Attrition — defined as the participants withdrawing from the study		✓	✓	✓	P
Attendance to intervention — the proportion of sessions attended		✓	✓	✓	S
Subjective feedback		✓	✓	✓	S

✓Recorded in that session, T_O_ onsite screening visit, T_1_ baseline assessment, T_2_ post-intervention assessment. *O* outcome measures, *D* Demographics, *P* Primary, *S* Secondary, *T* Tertiary

#### Biomarkers

As part of the baseline and post-training assessments, participants will undergo a blood draw at the Landon Center on Aging. The following biomarkers of neurodegeneration, inflammation, cellular senescence, and epigenetic dysregulation will be extracted from blood samples (serum/plasma): neurofilament-light (NfL), brain-derived neurotrophic factor (BDNF), glial fibrillary acidic protein (GFAP), C-reactive protein (CRP), interleukin-6 (IL-6), interleukin-10 (IL-10), tumor necrosis factor-alpha (TNF-alpha), interferon-gamma (IFN-gamma), and C-X-C motif chemokine ligand 9 (CXCL9) [23, 29–32]. In addition, miRNA profiling will be performed through miRNA-sequencing [29, 33].

#### Comprehensive frailty assessment

Following the blood draw, participants will complete a comprehensive assessment of frailty-related measures. The primary outcome of the study will be the Evaluative Frailty Index for Physical Activity (EFIP) [34]. The EFIP was designed to granularly assess the effects of physical activity and exercise interventions on frailty levels [34]. It evaluates 50 health-deficit items relating to multiple components of frailty (i.e., physical, psychological, social functioning, and general health) [34]. The EFIP has proven validity and reliability and has shown responsiveness to exercise programs involving motor-cognitive rehabilitation [34].

As secondary outcomes, we will assess quality of life by means of the 54-item Multiple Sclerosis Quality-of-Life questionnaire (MSQoL-54) [35]. We will also evaluate fall risk through the Physiological Profile Assessment (PPA) [36]. This comprehensive test evaluates multiple physiological factors to determine an individual’s risk of falls and has been widely used in older adults and in pwMS. Measuring quality of life and fall risk will provide insights into whether our exercise program is promising in improving these factors that are often seen as a consequence of frailty [37]. All frailty-related data will be collected by a single research assistant highly trained in conducting physical function assessments.

#### Feasibility outcomes

The following outcomes, based on the guiding principles for feasibility studies [38], will be used to examine the feasibility of the multimodal exercise training program: (a) participant recruitment, i.e., number of consented individuals/number of approached individuals; (b) retention rate, i.e., number of participants completing the intervention/number of participants enrolled at baseline; (c) appropriateness of data collection and training procedures, as quantified through the percentage of missing data for analysis purposes and through the number of training sessions completed and time spent in training (i.e., adherence); (d) participant safety, i.e., number of adverse and serious adverse outcomes throughout the study and self-reported pain, assessed by the numeric pain rating scale [39], during each training session; (e) evaluation of resources, i.e., difference between proposed and actual research timeline; and (f) user engagement, as assessed through the Study Participant Feedback Questionnaire (SPFQ) [40, 41].

### Randomization and blinding

After the baseline assessment, study participants will be randomized to one of the two groups using pre-established randomization codes devised by the biostatistician. Consistent with the CONSORT guidelines, the procedures of sequence generation, allocation concealment, and group allocation will be implemented in a blinded and independent manner to ensure proper research design adherence [42, 43].

### Study groups

The participants will be randomly assigned to one of two groups: the intervention group or the control group.

#### Intervention group (multimodal exercise)

The multimodal exercise training group will attend training sessions three times per week for a duration of 6 weeks, resulting in a total of 18 training visits. Each training visit will be conducted for a duration of 1 h. The training sessions take place in the Mobility Core Laboratory at the University of Kansas Medical Center. Participants will undergo a structured training protocol involving virtual reality treadmill training (VRTT) and evidence-based resistance training (RT) [44, 45]. The training session will consist of 15 min of VRTT, followed by 30 min of progressive RT, followed by another 15 min of VRTT. The interval training design was chosen to induce acute neuromuscular fatigue through RT [46, 47] before the final bout of VRTT. In addition, this protocol was specifically designed to counteract frailty in pwMS by targeting multiple domains of frailty, such as slowness, cognition, muscle weakness, and fatigue (Table 3).

**Table 3:** The multimodal exercise training program

**Exercise components**	**Duration**	**Description**	**Target (frailty domains)**^**a**^
1) VR treadmill training	15 min	Treadmill walking with interactive virtual environment	• Slowness • Cognition
2) RT	30 min	Seated leg press, seated knee extensions, seated chest press, seated lat pulldowns	• Muscle weakness • Shrinkage
3) VR treadmill training	15 min	Treadmill walking with interactive virtual environment	• Slowness, cognition • Exhaustion

*VR* Virtual reality, *RT* Resistance training

^a^The multimodal exercise program will also inherently target the “inactivity” component of frailty

As part of VRTT, participants will walk on a research-grade treadmill while navigating a virtual environment projected on a TV screen while receiving feedback from the system [8, 48]. The virtual environment will consist of obstacles and distractors found along different pathways and corridors and resembling challenging everyday life activities. Navigating through this environment will require coordinating walking behavior to negotiate the virtual obstacles. The level of cognitive-motor training will be individualized according to the participant’s level of performance. Overground gait speed will be assessed prior to the intervention and every 2 weeks thereafter and will be used to set the treadmill speed. More specifically, the treadmill speed will be set to 60–80% of the overground gait speed. This will help tailor the training to the individual’s current state and minimize fatigue problems. If participants are unable to complete 15 min of continuous walking, walking duration will be progressively increased in 1–3-min bouts until the prescribed time is reached. To maximize safety, participants will wear a harness during the treadmill training. In addition to increasing gait speed, the cognitive component of training will also be tailored to each participant and progress throughout the study. This will be achieved by manipulating the number, size, and shape of obstacles and distractors (e.g., cars and people passing by), as well as the frequency, speed, and direction at which obstacles appear within the virtual obstacle course.

The RT component will consist of performing evidence-based exercises involving both the upper (i.e., seated chest press and lat pulldowns) and lower body (i.e., seated leg press and knee extensions), as fully outlined in Table 3. Sitting RT exercises involving weight machines (i.e., closed kinetic chains) rather than free weights were chosen for safety reasons, as recommended by current guidelines on RT for frail individuals [16] and for pwMS [49]. All RT exercises will be supervised by a trained physical therapist. The estimated 1-repetition maximum (1-RM) of all RT exercises will be evaluated using validated standard procedures for untrained individuals [50] prior to the intervention. Specifically, the 1-RM of the different exercises will be estimated during the first training session by selecting a weight that allows participants to execute between 7 and 10 repetitions with maximum effort for each exercise. The formula described by Braith [50] will then be applied to estimate the 1-RM. At the beginning of training, participants will initially perform two sets of 8–12 repetitions for each exercise at 30% of the 1-RM and slowly progress up to 80% (endpoint: volitional fatigue) [16]. For each exercise, the weights will be increased upon the successful execution of at least 2 consecutive sets of 12 repetitions. It is anticipated that participants should be able to achieve a 10% increase each week, reaching 80% of their 1-RM during the 6th week of training (i.e., week 1: 30% 1-RM; week 2: 40% 1-RM; week 3: 50% 1-RM; week 4: 60% 1-RM; week 5: 70% 1-RM; week 6: 80% 1-RM). Recovery periods of up to 2 min will be allowed between sets and exercises.

#### Control group (waitlist)

Participants randomized to the control group will keep receiving their usual treatment over the 6-week study period, and they will become eligible to receive the exercise intervention only after the post-training assessments (Visit 3). No changes in prescribed medications or in lifestyle will be imposed; however, participants will be asked to refrain from participating in other exercise trials (same for participants in the intervention group) for the whole duration of the study. This passive waitlist control group was chosen, rather than an active control, to provide proof-of-concept data in this early phase of research.

### Statistical methods

Descriptive statistics (frequency/percent or mean/SD) will be mainly used to summarize participants’ characteristics and group outcomes. The intervention will be considered feasible if the retention rate and the proportion of completed sessions are more than 80%. The means and 95% CIs for pre-post changes in the primary (i.e., EFIP), secondary (i.e., quality of life and fall risk), and tertiary outcomes (i.e., biomarkers) will be estimated. The Wilcoxon rank-sum test will be used for comparing the pre-post changes in EFIP (between Visits 2 and 3), as well as in the secondary and tertiary outcomes, between the two groups. For this pilot study, participants without post-intervention data will be considered to have had no change in outcomes. Analyses based on subjects with complete data will also be conducted as sensitivity analyses. In addition, we will conduct Spearman correlation analyses to explore the relationship between biomarkers and EFIP at baseline. This analysis will allow us to better understand which biomarkers may be more strongly related to frailty in pwMS. A *p*-value ˂ 0.05 will be used to guide the statistical interpretation of all analyses. No control for multiple testing will be considered for this pilot study.

### Data and safety monitoring

The present research study will adhere to the guidelines for data and safety monitoring boards (DSMBs). The members of the DSMB will receive regular data reports at a schedule agreed upon by the members. In addition to the comprehensive evaluation of the study protocol, the DSMB will also meticulously assess the trial’s progress, encompassing factors such as recruitment stratified by ethnicity and sex, protocol deviations, adverse events, data integrity, attrition, and research outcomes, and subsequently offer suitable recommendations. Moreover, the study team will actively participate in DSMB meetings, which will be convened at least once/twice annually throughout the entirety of the study’s duration.

### Confidentiality

To ensure the utmost confidentiality of participants, some procedures, including informed consent, paperwork, and screening, will be implemented within the University of Kansas Medical Center. Each participant will be assigned a unique study identification number, and all study forms will be de-identified. Upon completion of the study, other researchers will be granted access to the de-identified study data. This will provide an opportunity for other researchers to pose additional inquiries in more detail.

### Dissemination plan

The results of the current research project will be published in a peer-reviewed journal, attributing authorship to individuals who have made substantial intellectual contributions to the study. The manuscripts will also undergo meticulous evaluation prior to submission for publication. Furthermore, the research findings may be disseminated through presentations at both domestic and international conferences.

## Discussion

The elevated risk of frailty in pwMS brings to light a significant challenge in managing this condition. The relationship between frailty and adverse clinical outcomes such as falls [2, 6] and the possibility of early onset of frailty in pwMS [4] emphasize the need for interventions that can mitigate the impact of frailty on the quality of life for pwMS in a timely manner [51, 52]. The current proposal builds on the well-established notion that exercise-based interventions play a significant role in restoring the health and well-being of pwMS [7], and it will be the first study to provide information concerning whether exercise interventions can modify frailty in pwMS.

The proposed project will address two main goals: examining the feasibility and preliminary efficacy of a multimodal exercise training program to reduce frailty in pwMS. The aim of the feasibility phase of the study is to assess the safety and acceptability of implementing a multimodal exercise training program for pwMS. We anticipate that at least 80% of the participants will successfully complete the exercise training with no serious adverse events during the training or assessment sessions. The preliminary efficacy phase of the study aims to investigate the feasibility of multimodal exercise training on frailty in pwMS. We expect that the participants in the exercise group will experience a greater reduction in the frailty index (EFIP) compared to the control group [53]. In this respect, the study will directly address methodological limitations that have plagued the field. Particularly, a recent Lancet review highlighted the importance of appropriately quantifying frailty prior to and following interventions (i.e., evaluating frailty as the primary study outcome) to better gauge their potential to reduce frailty, rather than exclusively measuring their effects on hallmarks of frailty such as muscle strength or gait speed [54].

### Potential limitations

The main potential problem for this project may consist of participant recruitment issues. Indeed, it is possible that frail pwMS may be unwilling to participate in an exercise-based training program. In addition to unwillingness to participate, injury or fatigue may prevent continued participation in the study. We will leverage our experience from our previous RCT [8] to incorporate stringent criteria for participants’ safety to minimize the risk of injury during the VRTT component of training. Standard and validated procedures for untrained individuals will be implemented to ensure safety during 1-RM testing and during RT. In the unlikely event that participants experience an injury, we will discontinue training and account for potential attrition through increased recruitment. We should also acknowledge that the relatively small sample size for the current study (*n* = 24, 12 participants in each group) may limit the statistical power to detect significant differences between the intervention and control groups, potentially leading to inconclusive findings. Additionally, the inclusion criteria restrict the study to pwMS with an Expanded Disability Status Scale (EDSS) score of up to 6.0, excluding individuals with more severe disabilities (and with potentially higher frailty levels [55]), which may limit the generalizability of the results to the broader MS population. Choosing a passive control group is a viable strategy for generating proof of concept data as intended in this feasibility RCT; however, it is important to acknowledge another limitation stemming from the lack of a robust placebo control group, which could potentially introduce bias due to heightened expectations for improvement among both participants and researchers in the intervention group.

### Future directions

The long-term vision of this pilot project is to move the field forward by providing comprehensive feasibility and proof of concept data, which will, in turn, be used to inform a future larger RCT investigation examining the efficacy of multimodal exercise training as a strategy to prevent/minimize frailty in pwMS. Additionally, it will establish the basis for our long-term objective of improving health span and quality of life in aging persons with or without MS. Future research should also focus on developing a comprehensive, multidisciplinary outlook on innovative strategies to address frailty’s impact and enhance the overall well-being of aging individuals with MS.

### Supplementary Information

**Additional file 1.** SPIRIT 2013 Checklist: Recommended items to address in a clinical trial protocol and related documents.

**Additional file 2.** The TIDieR (Template for Intervention Description and Replication) Checklist: Information to include when describing an intervention and the location of the information.

## Data Availability

Not applicable.

## Abbreviations

1-RM: One-repetition maximum
BDNF: Brain-derived neurotrophic factor
CRP: C-reactive protein
CXCL9: C-X-C motif chemokine ligand 9
DSMB: Data and Safety Monitoring Board
EDSS: Expanded Disability Status Scale
EFIP: Evaluative Frailty Index for Physical Activity
GFAP: Glial fibrillary acidic protein
IFN-gamma: Interferon-gamma
IL-10: Interleukin-10
IL-6: Interleukin-6
MS: Multiple sclerosis
MSQoL-54: 54-item Multiple Sclerosis Quality-of-Life questionnaire
NfL: Neurofilament light
PPA: Physiological profile assessment
PwMS: People with multiple sclerosis
RCT: Randomized clinical trial
RT: Resistance training
SPQF: Study Participant Feedback Questionnaire
TNF-alpha: Tumor necrosis factor-alpha
VRTT: Virtual reality treadmill training
